# Incidence and prognostic implications of PSA relapse after radical radiotherapy for prostate cancer: a population‐based study

**DOI:** 10.1111/bju.70148

**Published:** 2026-01-15

**Authors:** Paolo Zaurito, Hans Garmo, Rolf Gedeborg, Mats Alhberg, Stefan Carlsson, Camilla Thellenberg, David Robinson, Pär Stattin, Marcus Westerberg

**Affiliations:** ^1^ Department of Surgical Sciences Uppsala University Uppsala Sweden; ^2^ Department of Molecular Medicine and Surgery Karolinska Institutet Stockholm Sweden; ^3^ Department of Diagnostics and Intervention Umeå University Umeå Sweden; ^4^ Department of Urology Skåne University Hospital Malmö Sweden; ^5^ Division of Experimental Oncology/Unit of Urology, URI Institution IRCCS Ospedale San Raffaele Milan Italy

**Keywords:** prostate cancer, radical radiotherapy, prostate‐specific antigen, relapse, biochemical recurrence, prostate cancer death

## Abstract

**Objective:**

To estimate risk of prostate‐specific antigen (PSA) relapse after radical radiotherapy (RT) for prostate cancer (PCa), and risk of PCa death after relapse according to Gleason score and time to relapse.

**Patients and Methods:**

Men in the National Prostate Cancer Register of Sweden who underwent primary radical RT in 2007–2024 were followed until 31 December 2024. Relapse was defined as a PSA level increase of ≥2 ng/mL above nadir (Phoenix criteria). Competing risk cumulative incidence analyses were used to estimate risk of PSA relapse and risk of PCa death after relapse according to Gleason score and time to relapse.

**Results:**

The 10‐year risk of relapse in 26 634 men treated with RT was 25% (95% confidence interval [CI] 24–25%). The 10‐year risk of PCa death after relapse was 35% (95% CI 33–37%). In men with relapse after >3 years the risk was 18% for Gleason score 6 and 19% for Gleason score 3 + 4, while in men with a relapse within 18 months the risk was 52% for Gleason score 4 + 3 and 75% for Gleason score 9–10. In men with a relapse at 1 year after RT there was a four‐fold higher risk of PCa death for men with Gleason score 9–10 compared to men with Gleason score 6 (86% vs 22%). In contrast, in men with a relapse at 10 years after RT there were little differences in risk of PCa death according to Gleason (14% vs 23%).

**Conclusion:**

In this population‐based study of RT for PCa, there was a wide range in the estimates of risk of PCa death after relapse in highly granular groups according to Gleason score and time to relapse. Notably, some estimates did not align with the European Association of Urology relapse risk group classification.

**Abbreviations**
ADT: androgen deprivation therapy
ARPI: androgen receptor pathway inhibitors
cT: clinical T stage
EAU: European Association of Urology
IQR: interquartile range
IT: Information Technology
NPCR: National Prostate Cancer Register
PCa: prostate cancer
PCBase: Prostate Cancer data Base Sweden
(EB)RT: (external beam) radiotherapy

## Introduction

There has been a wide range in risk of PSA relapse, aka biochemical recurrence, after radical radiotherapy (RT) for prostate cancer (PCa), ranging from 19% to 50% at 5 years after RT in previous studies [1, 2, 3, 4, 5]. However, to what extent these studies are representative of current clinical practice is unclear as the studies were conducted in 1988–2002, and most of them were single‐centre studies [1, 2, 3, 4, 5]. In a recent population‐based study in Stockholm, Sweden of 5947 men, the risk of relapse within 15 years after RT ranged from 18% in men with D’Amico low‐risk cancer to 36% in men with high‐risk PCa [6].

The risk of death from PCa after a relapse in men treated with RT is influenced by Gleason score at biopsy and time from RT to relapse [7, 8, 9, 10, 11, 12, 13]. According to the European Association of Urology (EAU) classification, a low‐risk relapse is defined as relapse >18 months after RT in men with Gleason score 6–7, and high‐risk relapse is defined as relapse within 18 months of RT or in men with Gleason score 8–10 [14, 15].

The aim of our study was to assess risk of PSA relapse after radical RT for PCa and risk of death from PCa after relapse according to Gleason score and time to relapse in a nationwide population‐based cohort.

## Patients and Methods

### Data Sources

The National Prostate Cancer Register (NPCR) of Sweden is a clinical cancer register with the primary aim to benchmark adherence to national guidelines [16]. In the Prostate Cancer data Base Sweden (PCBase), the NPCR has been linked to other health care registers and demographic databases including The National Patient Register, The Prescribed Drug Register, the socioeconomic database Longitudinal Integrated Database for Health Insurance and Labor Market Studies (LISA), The Total Population Register, and The Cause of Death Register [17]. In the PCBase Xtend longitudinal data from healthcare Information Technology (IT) systems on serum PSA, prostate MRI, prostate biopsies, use of androgen deprivation therapy (ADT) and androgen receptor pathway inhibitors (ARPI) has been retrieved from all 21 regions in Sweden and linked with the PCBase [18]. The Swedish Ethical Review Authority approved the study. The study adheres to the Strengthening the Reporting of Observational Studies in Epidemiology (STROBE) reporting guideline.

### Study Population

Men with PCa who underwent primary radical RT in 2007–2024, registered in the NPCR and residing in regions where longitudinal data on PSA had been added to PCBase Xtend at the date of RT were included. Radical RT included conventionally fractionated, moderately hypofractionated, ultra hypofractionated external beam RT (EBRT), and conventionally fractionated EBRT combined with brachytherapy. Radiation doses for the protocols are provided in Table S1. Men were excluded if data on PSA, Gleason score, or clinical T stage (cT) before RT was missing, orchidectomy had been performed before the end of RT, metastases were detected on imaging, or imaging had not been performed despite recommendation in the Swedish Prostate Cancer Guidelines that recommend that men with PSA level ≥20 ng/mL, cT3–4, or Gleason score ≥8 undergo imaging.

### Data Extracted

We extracted age at RT, Gleason score at biopsy, start date, duration and dose of RT, PSA level at diagnosis and during follow‐up, and clinical TNM stage. We used a modification of the National Comprehensive Cancer Network (NCCN) risk classification [19]: low‐risk (cT1–2, Gleason score 6, and PSA level <10 ng/mL), favourable intermediate‐risk (cT1–2, Gleason score 7 [3 + 4], and PSA level <10 ng/mL or Gleason score 6 and PSA level ≤10 to <20 ng/mL), unfavourable intermediate‐risk (cT1–2, Gleason score 7 [4 + 3] and PSA level <20 ng/mL or Gleason score 7 [3 + 4] and PSA level ≤10 to <20 ng/mL), high‐risk (cT1–2, Gleason score 8–10 or PSA level ≥20 ng/mL), locally advanced (any PSA level, any Gleason score, cT3–4).

Use of ADT including GnRH agonists, bicalutamide, and ARPI (i.e., abiraterone, darolutamide, apalutamide, enzalutamide), was assessed by analysis of entries in The Prescribed Drug Register and by use of data in regional healthcare IT systems. In Sweden, adjuvant monotherapy with bicalutamide for 2–3 years after radical RT is recommended for men with unfavourable intermediate‐risk and high‐risk PCa. Further, recent guidelines recommend that men with very‐high risk PCa receive adjuvant therapy with a GnRH agonist for 3 years and abiraterone and prednisolone for 2 years [20]. Use of ciprofloxacin was extracted from The Prescribed Drug Register.

Life expectancy at date of RT was calculated based on age and comorbidity, quantified by a Multidimensional Diagnosis‐based Comorbidity Index and a Drug Comorbidity Index, at date of RT using previously described methods [21, 22, 23, 24]. Date of migration was extracted from The Total Population Register and date and cause of death from The Cause of Death Register.

### Outcomes

Relapse was defined according to the Phoenix definition of relapse by the American Society for Therapeutic Radiology and Oncology (ASTRO) and the Radiation Therapy Oncology Group (RTOG) as a PSA level ≥2 ng/mL above nadir PSA level, i.e., the lowest PSA level observed so far after RT [25]. We did not include PSA values potentially affected by acute prostatitis (assessed by use of ciprofloxacin within ±28 days of the date for measurement of PSA) in the assessment of PSA ≥nadir +2 ng/mL.

Androgen deprivation therapy was considered adjuvant if it started before the date of the first PSA measurement after the end of RT and ended when there were no entries for ADT after 189 days after the end of the previous entry of ADT (based on defined daily dose and grace period considered as wash‐out time). Non‐adjuvant ADT was defined as ADT that started after the first PSA measurement after RT in men who did not receive adjuvant ADT, and in men who received adjuvant ADT it was defined as ADT that started after the end of the adjuvant ADT as defined above. Men who underwent radical prostatectomy or started non‐adjuvant ADT prior to PSA relapse according to the above definition were not included in the relapse group.

We used the EAU relapse risk groups; EAU low‐risk: relapse >18 months after RT and Gleason score 6–7, and EAU high‐risk: relapse within 18 months after RT or Gleason score 8–10 [14].

### Sensitivity Analyses

We performed a sensitivity analysis where we ignored the use of ciprofloxacin and included all PSA levels in the assessment of the Phoenix criteria. We also performed sensitivity analyses using 90‐ and 365‐day wash‐out times to determine end of adjuvant ADT, and in an additional sensitivity analysis, we used a nadir cut‐off of 1 ng/mL for men on adjuvant ADT.

### Statistical Analysis

Follow‐up began at the end of RT and ended at one of the following events: relapse, start of treatment (radical prostatectomy or non‐adjuvant ADT without documented PSA relapse), death from PCa or other causes, or until 31 December 2024. We computed the cumulative incidence proportion of relapse and other competing risks according to risk category, life expectancy at diagnosis, and calendar period of RT (2007–2015 and 2016–2024). Men who emigrated or moved to a region where data on longitudinal PSA level and ADT data were not available were censored at the time they moved.

In men with relapse, we computed the proportion of men who received ADT and/or ARPI within 6 months from relapse and cumulative incidence proportion of death from PCa and other causes according to Gleason score, time to relapse, PSA doubling time, calendar period of relapse, and use of adjuvant ADT at relapse. We also estimated the absolute risk of death from PCa by use of a parametric discrete‐time competing‐risk models based on Gleason score, time to relapse, and PSA doubling time as continuous variables modelled using three‐dimensional thin‐plate regression splines with shrinkage [26]. For men who experienced a relapse, follow‐up began at the date of relapse and ended on the date of death, migration, or on 31 December 2024.

All analyses were performed using R, version 4.3.2 (R Foundation for Statistical Computing, Vienna, Austria). All cumulative incidence estimates were accompanied by 95% CIs.

## Results

### Study Population

There were 26 634 men with non‐metastatic PCa registered in the NPCR who had received primary radical RT in 2007–2024 and had data on PSA at RT, T stage at RT, Gleason score in biopsy, and data on PSA and ADT during follow‐up (Fig. S1).

### Characteristics at RT

The median (interquartile range [IQR]) age at RT was 71 (66–750 years) (Table 1). The median (IQR) PSA level at diagnosis ranged from 7 (5–9) ng/mL in men with low/favourable intermediate‐risk PCa to 15 (7–32) ng/mL in men with locally advanced PCa. The most common type of RT was conventionally fractionated EBRT (*n* = 9607 [36%]), followed by moderately hypofractionated EBRT (*n* = 8056 [30%]) and conventionally fractionated EBRT + brachytherapy (*n* = 4568 [18%]).

**Table 1:** Baseline characteristics of the 26 634 men treated with radical RT in the NPCR of Sweden.

Characteristic	Risk category before start of RT*		
	Low‐/favourable intermediate‐risk	Unfavourable intermediate‐/high‐risk	Locally advanced
**Number of men**, *n* (%)	8944 (100)	13 141 (100)	4549 (100)
**Age at RT, years**			
Median (IQR)	69 (65–73)	71 (67–75)	70 (65–75)
<60, *n* (%)	706 (8)	726 (6)	328 (7)
60–70, *n* (%)	4460 (50)	5118 (39)	2000 (44)
>70, *n* (%)	3778 (42)	7297 (56)	2221 (49)
**Year of RT**, *n* (%)			
2007–2011	1600 (18)	1780 (14)	893 (20)
2012–2015	1788 (20)	2629 (20)	1009 (22)
2016–2020	3678 (41)	5562 (42)	1778 (39)
2021–2024	1878 (21)	3170 (24)	869 (19)
**Civil status**, *n* (%)			
No partner	3174 (35)	4711 (36)	1696 (37)
Partner	5770 (65)	8430 (64)	2853 (63)
**Educational level** ^†^, *n* (%)			
Low	2376 (27)	3864 (29)	1382 (30)
Intermediate	3891 (44)	5487 (42)	1856 (41)
High	2677 (30)	3790 (29)	1311 (29)
**Income**, *n* (%)			
<Q1	1792 (20)	2865 (22)	977 (21)
Q1–Q2	1923 (22)	2818 (21)	938 (21)
Q2–Q3	2549 (28)	3768 (29)	1372 (30)
>Q3	2680 (30)	3690 (28)	1262 (28)
**Type of RT**, *n* (%)			
Conventionally fractionated EBRT	2808 (31)	4871 (37)	1928 (42)
Moderately hypofractionated EBRT	2452 (27)	4136 (31)	1468 (32)
Ultra hypofractionated EBRT	2298 (26)	1852 (14)	253 (6)
Conventionally fractionated EBRT plus brachytherapy	1386 (15)	2282 (17)	900 (20)
**PSA level before RT, ng/mL**			
Median (IQR)	7 (5–9)	12 (7–20)	15 (7–32)
<10, *n* (%)	7796 (87)	5011 (38)	1662 (37)
10–20, *n* (%)	1148 (13)	4934 (38)	1123 (25)
>20, *n* (%)	0 (0)	3196 (24)	1764 (39)
**T stage before RT**, *n* (%)			
T1	5631 (63)	6007 (46)	0 (0)
T2	3313 (37)	7134 (54)	0 (0)
T3	0 (0)	0 (0)	4388 (96)
T4	0 (0)	0 (0)	161 (4)
**Gleason score before RT**, *n* (%)			
6	3589 (40)	439 (3)	256 (6)
7 (3 + 4)	5355 (60)	3000 (23)	934 (21)
7 (4 + 3)	0 (0)	4469 (34)	1095 (24)
8	0 (0)	2499 (19)	846 (19)
9–10	0 (0)	2734 (21)	1418 (31)
**Life expectancy at RT, years**			
Median (IQR)	15 (13–19)	14 (11–17)	14 (11–18)
≤15, *n* (%)	4282 (48)	8099 (62)	2545 (56)
>15, *n* (%)	4662 (52)	5042 (38)	2004 (44)

Abbreviations: Q1, first quartile; Q2, second quartile; Q3, third quartile.

* Risk categories were defined based on NPCR's modification of National Comprehensive Cancer Network (NCCN) risk categorisation: (i) low‐risk category; cT1–2, Gleason score 6 and PSA level <10 ng/mL, (ii) favourable intermediate‐risk category; cT1–2, Gleason score 7 (3 + 4) and PSA level <10 ng/mL or Gleason score 6 and PSA level ≤10 to <20 ng/mL, (iii) unfavourable intermediate‐risk category; cT1–2, Gleason score 7 (4 + 3) and PSA level <20 ng/mL or Gleason score 7 (3 + 4) and PSA level ≤10 to <20 ng/mL, (iv) high‐risk category; cT1–2 and Gleason score 8–10 or PSA level ≥20 ng/mL, (v) locally advanced category; any PSA level, any Gleason, cT3–4.

^†^ Low is <10 years (mandatory school), intermediate is 10–12 years (high school), high is more than 12 years of education (university).

### Incidence of Relapse

**Fig. 1:**
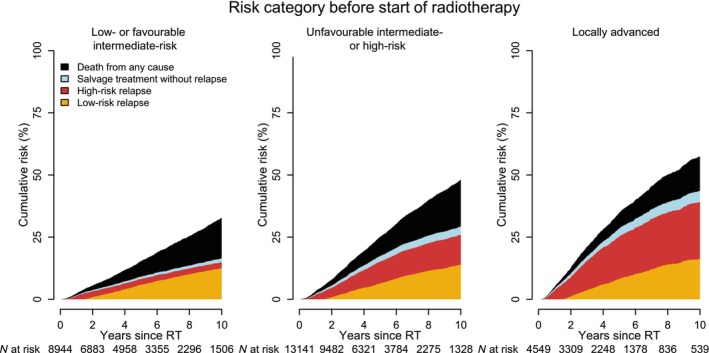
Cumulative incidence proportion of relapse and treatment performed after radical RT among 26 634 men registered in PCBase 5 Xtend according to risk category at diagnosis. Salvage treatments without relapse are radical prostatectomy or ADT without evidence of PSA relapse in our data.

The 10‐year risk of relapse was 25% (95% CI 24–25%) overall and ranged from 15% (95% CI 14–15%) in men with low/favourable intermediate‐risk PCa to 39% (95% CI 37–41%) in men with locally advanced PCa (Fig. 1). The 10‐year risk of relapse was similar in men with life expectancy above and below 15 years (26% vs 23%), whereas there was a two‐fold difference in risk of death from other causes (12% vs 29%; Fig. S2). The 5‐year risk of relapse was somewhat lower in 2016–2024 compared to 2007–2015 (12% vs 17%).

### Characteristics of Men with PSA Relapse

Of the 4024 men with a PSA relapse after RT, 819 (20%) had a relapse within 18 months after RT, 1021 (25%) between 18 and 36 months, and 2184 (55%) relapsed after 36 months (Table 2). Men with a relapse within 18 months after RT had a shorter PSA doubling time, higher PSA level at nadir, and higher PSA level at relapse compared to men with a relapse after 36 months.

**Table 2:** Baseline demographic and cancer characteristics of the 4024 men who had a PSA relapse after radical RT.

Characteristic	Time interval from RT to relapse		
	<18 months	18–36 months	>36 months
Number of men, *n* (%)	819 (100)	1021 (100)	2184 (100)
**Age at relapse, years**			
Median (IQR)	69 (64–74)	72 (67–76)	75 (70–79)
<60, *n* (%)	83 (10)	72 (7)	49 (2)
60–70, *n* (%)	382 (47)	373 (37)	535 (24)
>70, *n* (%)	354 (43)	576 (56)	1600 (73)
**Nadir PSA level, ng/mL**			
Median (IQR)	1.6 (0.4–4.0)	0.2 (0.1–0.8)	0.1 (0.1–0.4)
<0.1, *n* (%)	90 (11)	454 (44)	1211 (55)
0.1–0.5, *n* (%)	143 (17)	242 (24)	542 (25)
≥0.5, *n* (%)	586 (72)	325 (32)	431 (20)
**PSA level at relapse, ng/mL**			
Median (IQR)	6 (4–13)	4 (3–6)	3 (2–4)
≤5, *n* (%)	301 (37)	687 (67)	1819 (83)
5–10, *n* (%)	261 (32)	225 (22)	225 (10)
≥10, *n* (%)	257 (31)	109 (11)	140 (6)
**PSA doubling time** *, **months**			
Median (IQR)	3 (2–4)	5 (4–7)	13 (9–20)
<9, *n* (%)	776 (95)	888 (87)	548 (25)
≥9, *n* (%)	43 (5)	133 (13)	1636 (75)
**EAU relapse risk group** ^†^, *n* (%)			
Low‐risk relapse	0 (0)	575 (56)	1471 (67)
High‐risk relapse	819 (100)	446 (44)	713 (33)
**Gleason score before RT**, *n* (%)			
6	121 (15)	109 (11)	350 (16)
7 (3 + 4)	195 (24)	234 (23)	586 (27)
7 (4 + 3)	171 (21)	232 (23)	535 (24)
8	128 (16)	167 (16)	309 (14)
9–10	204 (25)	279 (27)	404 (18)
**Risk category before RT** ^‡^, *n* (%)			
Low‐risk/favourable intermediate‐risk	193 (24)	165 (16)	491 (22)
Unfavourable intermediate‐risk/ high‐risk	364 (44)	512 (50)	1087 (50)
Locally advanced	262 (32)	344 (34)	606 (28)
**Relapse while on adjuvant ADT** ^§^, *n* (%)			
No	471 (58)	710 (70)	2091 (96)
Yes	348 (42)	311 (30)	93 (4)
Bicalutamide monotherapy	177 (22)	263 (26)	71 (3)
GnRH monotherapy	81 (10)	26 (3)	13 (1)
Bicalutamide and GnRH	87 (11)	21 (2)	9 (0)
Orchidectomy	3 (0)	1 (0)	0 (0)

* PSA doubling time was computed using all PSA values from nadir to PSA at relapse.

^†^ Relapse risk groups according to EAU guidelines: low‐risk relapse (time interval from RT to PSA relapse >18 months and biopsy Gleason score 6–7) and high‐risk relapse (time interval to PSA relapse within 18 months or biopsy Gleason score 8–10).

^‡^ Risk categories were defined based on NPCR's modification of National Comprehensive Cancer Network (NCCN) risk categorisation: (i) low‐risk category; cT1–2, Gleason score 6 and PSA level <10 ng/mL, (ii) favourable intermediate‐risk category; cT1–2, Gleason 7 (3 + 4) and PSA level <10 ng/mL or Gleason score 6 and PSA level ≤10 to <20 ng/mL, (iii) unfavourable intermediate‐risk category; cT1–2, Gleason score 7 (4 + 3) and PSA level <20 ng/mL or Gleason score 7 (3 + 4) and PSA level ≤10 to <20 ng/mL, (iv) high‐risk category; cT1–2 and Gleason score 8–10 or PSA level ≥20 ng/mL, (v) locally advanced category; any PSA level, any Gleason score, cT3–T4.

^§^ Men were considered to be affected by adjuvant ADT if they had an orchidectomy before relapse or if the relapse occurred before the date of the most recent dose of bicalutamide or GnRH before relapse + the dose duration (based on defined daily dose) + 189 days.

### Treatment at Relapse

At the time of relapse, 3272 (81%) men were not on adjuvant ADT (Table 2). Of these, 1284 (40%) men received ADT only, and in those with a relapse in 2016–2024 38% received ADT only and 4% received an ARPI (Table S2). In contrast, 20% among those with a relapse in 2016–2024 while on adjuvant ADT received an ARPI in addition to ADT. The proportion of men who received additional treatment was higher in men with high Gleason scores and short times to relapse and increased between 2007–2015 and 2016–2024.

### Risk of PCa Death after Relapse

**Fig. 2:**
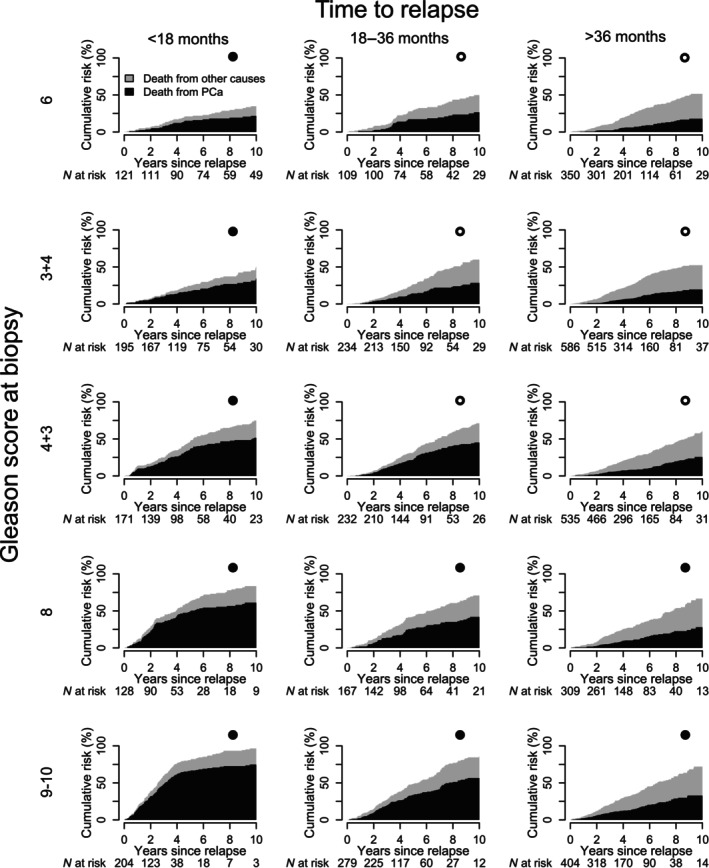
Cumulative incidence proportion of death from PCa and other causes in 4024 men with relapse after radical RT. Filled circles denote EAU high‐risk relapse groups and empty circles denote EAU low‐risk relapse groups.

The 10‐year risk of PCa death was 35% (95% CI 33–37%) after relapse and ranged from 20% for Gleason score 6 to 52% for Gleason score 9–10 (Fig. 2). There was a substantial difference in risk groups according to combinations of time to relapse and Gleason score. In men with relapse after >3 years, risk of PCa death was 18% for Gleason score 6 and 19% for Gleason score 3 + 4, while in men with a relapse within 18 months, risk was 52% for Gleason score 4 + 3 and 75% for Gleason score 9–10.

**Fig. 3:**
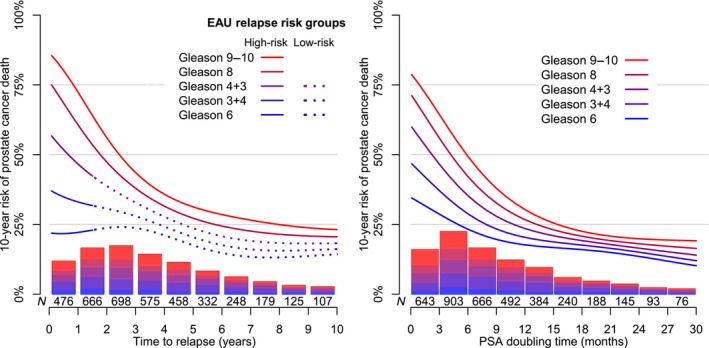
Cumulative incidence proportion of death from PCa within 10 years from relapse. Estimates were obtained by use of a discrete time parametric model. Death from other causes was considered a competing risk. Bars indicate the distribution of men with relapse according to Gleason score and time to relapse (left) or PSA doubling time (right).

There was a wide range in the 10‐year risk of PCa death according to Gleason score for men with an early relapse but little differences according to Gleason score for men with a late relapse (Fig. 3). In men with a relapse at 1 year after RT there was a four‐fold higher risk of PCa death for men with Gleason score 9–10 (86%) compared to men with Gleason score 6 (22%). In contrast, in men with a relapse at 10 years after RT the differences according to Gleason score were smaller (14–23%). There was a continuous decrease in risk up to well over 4 years after RT.

A similar pattern was observed for PSA doubling time (Fig. 3). Men with PSA doubling time of 6 months had a two‐fold higher risk of PCa death for men with Gleason score 9–10 (70%) compared to men with Gleason score 6 (34%), with smaller differences according to Gleason score for longer doubling times, e.g., (16–23%) at 18 months.

Men who had a relapse in 2016–2024 had a lower risk of PCa death compared to men with a relapse in 2007–2015 (16% vs 25% at 5 years after relapse) and men who had a relapse while on adjuvant ADT had a higher risk of PCa death compared to men not on adjuvant ADT (60% vs 31% at 10 years after relapse; Figs S3 and S4).

### Sensitivity Analyses

The results across all sensitivity analyses were in accordance with our main analysis (Table S3).

## Discussion

In this large population‐based study, risk of PSA relapse was 25% within 10 years after radical RT and ranged from 15% to 39% according to risk category before treatment. The 10‐year risk of PCa death for men who had a relapse after >3 years was 18% for Gleason score 6 and 19% for Gleason score 3 + 4, while in men with a relapse within 18 months this risk was 52% for Gleason score 4 + 3 and 75% for Gleason score 9–10. There was a continuous decrease in risk of PCa death with longer time to relapse and increasing PSA doubling time. Risk of death from PCa was somewhat lower in the late vs early calendar period.

### Strength and Limitations

The main strength of our study is the population‐based design and the large sample size that enabled us to estimate the risk of relapse after RT and PCa death after relapse in highly granular categories according to Gleason score and time to relapse. The NPCR, the clinical cancer register in Sweden, captures >98% of men diagnosed with PCa in Sweden compared with the Cancer Register, to which reporting is legally mandated [27]. The NPCR also contains comprehensive data on diagnostic evaluations including imaging and primary therapy, and The Prescribed Drug Register is nationwide population‐based register with high validity [28]. Longitudinal data on PSA and ADT delivered via hospital clinics during follow‐up are an enrichment of the dataset in PCBase Xtend. Life expectancy, critical for treatment selection, was estimated using two new comorbidity indices, which predict death better than the commonly used Charlson Comorbidity Index [21, 22, 23, 24].

Our study has some limitations. The inclusion period was 17 years, and risk of PCa death was somewhat lower in the second half of the study period so our estimates of risk of PCa death are likely to be somewhat higher than what can be expected in men who undergo RT today. Data on end of adjuvant ADT and start of non‐adjuvant ADT were not available, so we estimated it based on patterns of entries of ADT and by use of a wash‐out period. Data on metastases at relapse were not available and there was no information on extracapsular extension on MRI prostate performed before RT.

### Previous Results of Risk of PSA Relapse after RT

Our 10‐year estimates for risk of relapse for low/favourable intermediate‐risk PCa (15%) and locally advanced PCa (39%) are in accordance with a previous Swedish study; in which the 15‐year risk of relapse after RT ranged from 18% for D’Amico low‐risk, 24% for intermediate‐risk, and 39% for high‐risk PCa [6].

### Interpretation and Previous Studies on Risk of Death after Relapse

In previous studies, Gleason score, time to relapse, and PSA doubling time were strong prognostic factors for PCa death after RT [7, 8, 9, 10, 11, 12, 13, 29]. In a recent meta‐analysis, the strongest prognostic factor for PCa death was a short time to relapse [11]. We were able to estimate risk of death from PCa and other causes for highly granular categories of combinations of Gleason score and time to relapse, as well as PSA doubling time. These factors were strongly associated with risk of PCa death. However, for late relapses and long PSA doubling times the influence of Gleason score on the risk of PCa death was minimal.

### Interpretation in Relation to EAU Relapse Risk Groups

To date, only the Stockholm study has reported risk of PCa death after RT according to the EAU relapse risk group classification and the 15‐year risk of PCa death was 24% in men with an EAU low‐risk relapse and 46% in men with an EAU high‐risk relapse [6].

Due to the large size of our cohort, we were able to estimate risk of PCa death in highly granular categories of combinations of Gleason score and time to relapse. We found differences in risk of PCa death between Gleason score 7 (3 + 4) and (4 + 3), which are not differentiated in the EAU relapse risk categorisation. Furthermore, we found a continuous decrease in risk of PCa death with longer time to relapse, extending well over 4 years and for the few men with a very long time to relapse there were only small differences according to Gleason score. Thus, the dichotomisation of time to relapse at 18 months discards important prognostic information.

### Different Prognostic Implications of Relapse after Radical RT and Radical Prostatectomy

In our recent study on radical prostatectomy, the 10‐year risk of PSA persistence or relapse was 34% and the 10‐year risk of PCa death for men with a PSA relapse was low, 2% in men with low‐risk relapse and 12% in men with high‐risk relapse [30]. In the present study, the 10‐year risk of relapse after RT was somewhat lower (25%), but the 10‐year risk of PCa death after relapse was five–10‐fold higher. Relapse after radical prostatectomy and RT thus carries entirely different clinical implications. At relapse after radical prostatectomy a major challenge is to avoid overtreatment, whereas after RT aggressive treatment at relapse is warranted for many men. However, only half of men in our study received treatment in the form of ADT within 6 months after relapse.

### Generalisability

We argue that the results on incidence and prognostic implications of PSA relapse after RT in our nationwide population‐based study are representative for men in other countries in which similar treatments are implemented.

## Conclusion

In this nationwide population‐based study, the 10‐year risk of relapse after radical RT was 25%, with a wide range according to PCa risk category. For men who relapsed, the risk of PCa death at 10‐year was 35% with a wide range according to combinations of Gleason score and time to relapse, which are not fully captured by the current EAU relapse risk classification.

## Funding

This project was supported by The Vetenskapsrådet Research Council (2022‐00544), The Cancerfonden Cancer Society (2022‐2051), Region Uppsala, and Uppsala University. The sponsors had no involvement with the planning, execution or completion of the study.

## Disclosure of Interests

The authors report no conflicts of interest.

## Disclaimer

Rolf Gedeborg is employed by the Medical Products Agency (MPA) in Sweden. The MPA is a Swedish Government Agency. The views expressed in this article may not represent the views of the MPA.

## Supporting information


**Fig. S1.** Study flow chart. Men with PCa treated with radical RT from 2007 to 2024 who met the inclusion criteria for the study.
**Fig. S2.** Cumulative incidence proportion of relapse after radical RT according to life expectancy at diagnosis. *Salvage treatment without relapse included radical prostatectomy and ADT without evidence of PSA relapse in our data.
**Fig. S3.** Risk of death from PCa and other causes after relapse according to time to relapse, Gleason score at biopsy and calendar period of relapse.
**Table S1.** Dose plan according to RT type.
**Table S2.** Proportion of treatment within 180 days after relapse in men who experienced a relapse after primary RT according to time to relapse, Gleason score at biopsy, type of adjuvant ADT, and calendar period of relapse.
**Table S3.** Sensitivity analyses.

## Data Availability

None. Additional Information: Explanation for why data not available: Data used for the present study have been extracted from PCBase. Data can be made available on a remote server upon request to the PCBase reference group, contact par.stattin@.uu.se. The code used for the analyses can be provided on request by marcus.westerberg@uu.se.
