# Supplementary material for: Incidence and prognostic implications of PSA relapse after radical radiotherapy for prostate cancer: a population‐based study
Source: BJU Int. 2026 Jan 15;137(4):629–38. doi: 10.1111/bju.70148 (PMC12962848; doi:10.1111/bju.70148)
Supplement: Supplementary file 1 — Fig. S1. Study flow chart. Men with PCa treated with radical RT from 2007 to 2024 who met the inclusion criteria for the study. Fig. S2. Cumulative incidence proportion of relapse after radical RT according to life expectancy at diagnosis. *Salvage treatment without relapse included radical prostatectomy and ADT without evidence of PSA relapse in our data. Fig. S3. Risk of death from PCa and other causes after relapse according to time to relapse, Gleason score at biopsy and calendar period of relapse. Table S1. Dose plan according to RT type. Table S2. Proportion of treatment within 180 days after relapse in men who experienced a relapse after primary RT according to time to relapse, Gleason score at biopsy, type of adjuvant ADT, and calendar period of relapse. Table S3. Sensitivity analyses. [file BJU-137-629-s001.docx]

# Supplementary figures

Supplementary Figure 1. Study flow chart. Men with prostate cancer treated with radical RT from 2007 to 2024 who met the inclusion criteria for the study.

**All men registered in NPCR who underwent any form of radiotherapy from 2007 to 2024
(n=40,280)**

**(n = 45,617)**

**Men included in the study
(n = 26,634)**

**All men registered in NPCR who underwent radical radiotherapy* from 2007 to 2024
(n = 34,691)**

**All men registered in NPCR who underwent radical radiotherapy from 2007 to 2024 with longitudinal PSA and GnRH data available
(n = 28,031)**

**Imaging**

Imaging not performed despite recommendation according to current guidelines (**n = 46**)

**Clinical features**

M1 **(n = 699)**

Prior orchiectomy **(n = 6)**

**Missing data**

Missing PSA at RT (**n = 103**)

Missing T stage at RT **(n = 395)**

Missing Gleason at RT **(n = 148)**

**PSA and GnRH data**

Longitudinal PSA and GnRH data not available at the date of radiotherapy **(n = 6660)**

**Type of radiotherapy**

Did not receive radical radiotherapy* **(n = 5589)**

**Abbreviations**: National Prostate Cancer Register (NPCR).

*Radical radiotherapy was defined as conventionally fractioned, moderately hypofractionated, ultra hypofractionated external beam radiotherapy (EBRT), or conventionally fractioned EBRT combined with brachytherapy.

Supplementary Figure 2. Cumulative incidence proportion of relapse after radical radiotherapy according to life expectancy at diagnosis. *Salvage treatment without relapse included radical prostatectomy and ADT without evidence of PSA relapse in our data.
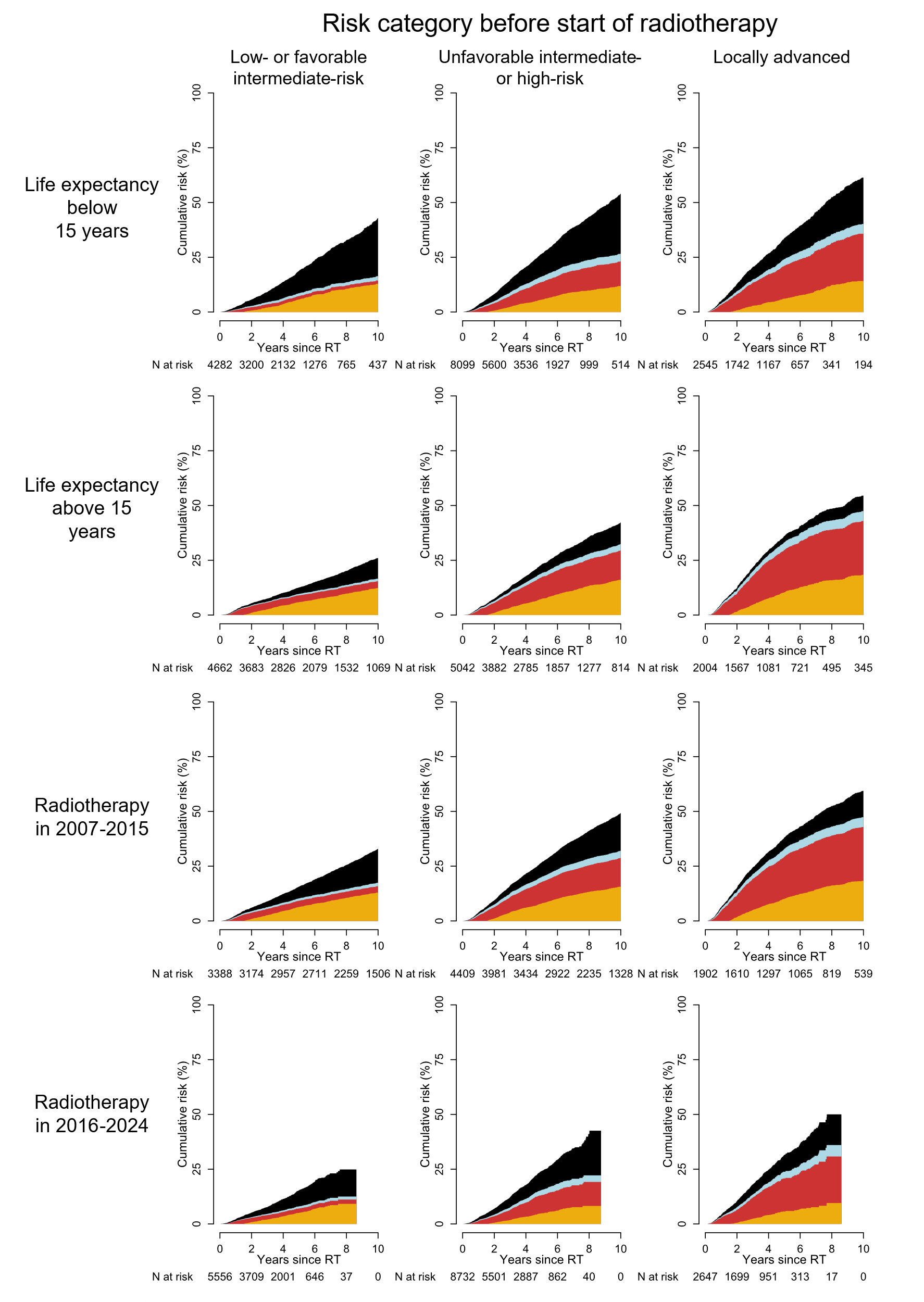


Supplementary Figure 3. Risk of death from prostate cancer and other causes after relapse according to time to relapse, Gleason score at biopsy and calendar period of relapse.


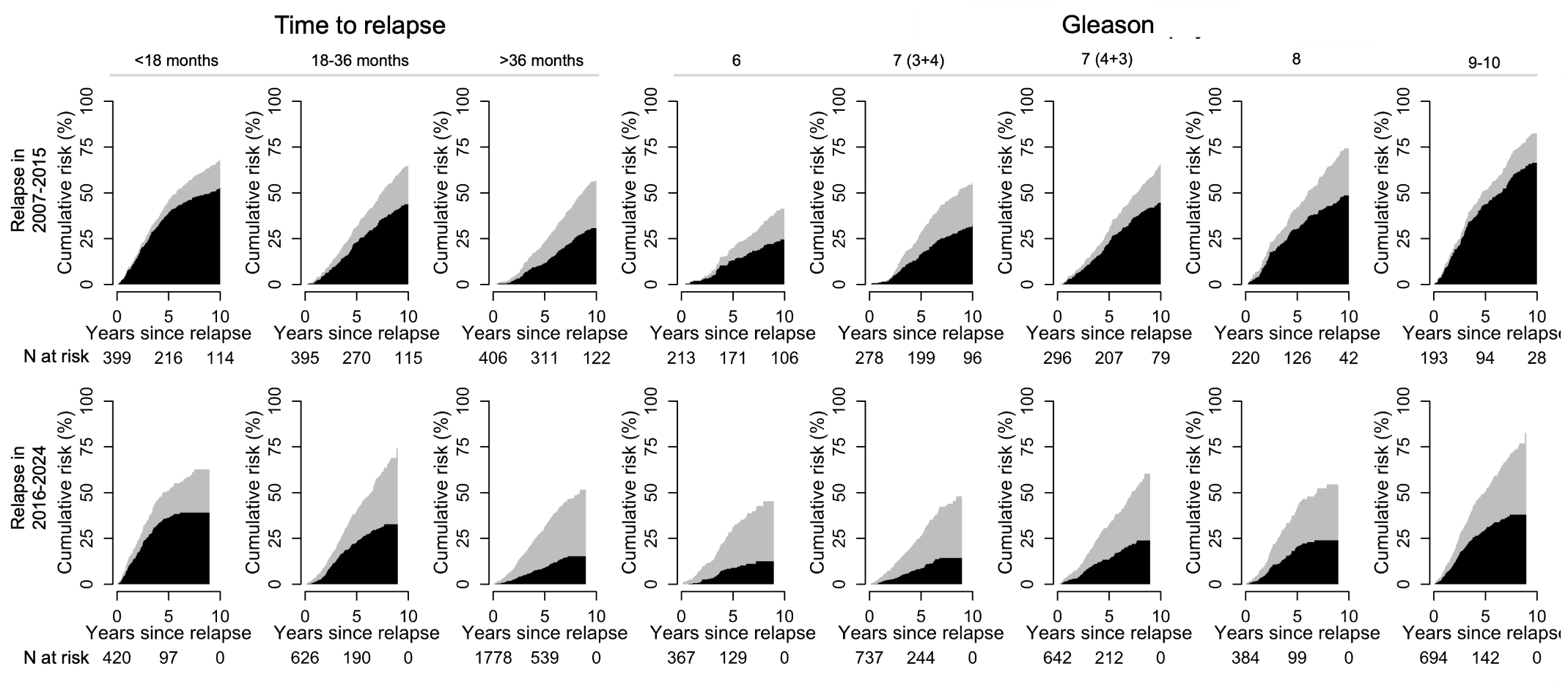


Supplementary Figure 4. Risk of death from prostate cancer and other causes after relapse according to whether the relapse occurred while the man was on adjuvant ADT or not, time to relapse and Gleason score at biopsy.


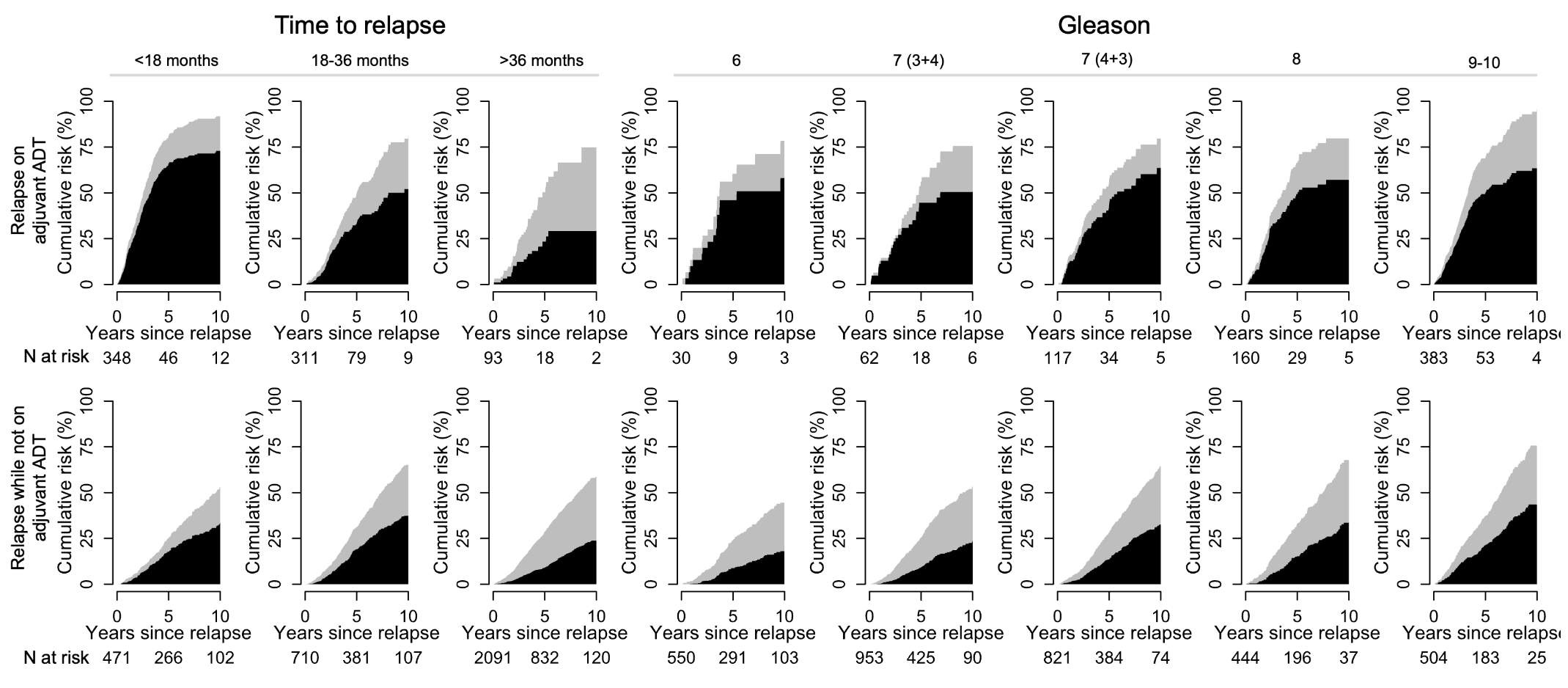


# Supplementary tables

Supplementary Table 1. Dose plan according to radiotherapy type.

| **Type** | **Dose** | **EQD2_3_** | **Number of men** | **Proportion (%)** |
| --- | --- | --- | --- | --- |
| CF-EBRT | 2 Gy to 74-77 Gy | 74-82 Gy | 131 | 0.5 |
|  | 2 Gy to 78 Gy |  | 8679 | 33 |
|  | 2 Gy to 80-82 Gy |  | 797 | 2.5 |
| MH-EBRT | 2.5 Gy to 72.5 Gy | 77-88 Gy | 3248 | 12 |
|  | 3 Gy to 60-66 Gy |  | 4808 | 18 |
| UH-EBRT | 6.1 Gy to 42.7-43.1 Gy | 77 Gy | 4403 | 16 |
| CF-EBRT+BT | 2 Gy to 50 Gy  + 10 Gy to 20 Gy | 101 Gy | 4568 | 18 |

**Abbreviations**:

Gy: Gray

EQD2_3_: equivalent total dose at 2 Gy per fraction using an alpha-beta ratio of 3 (1–3).

EBRT: external-beam radiotherapy

CF-EBRT: conventionally fractioned EBRT

MH-EBRT: moderately hypofractioned EBRT

UH-EBRT: ultra hypofractioned EBRT

CF-EBRT+BT: CF-EBRT combined with brachytherapy

| **Relapse in 2007-2015** | | | | | | | | | | | |
| --- | --- | --- | --- | --- | --- | --- | --- | --- | --- | --- | --- |
| **Type of adjuvant ADT** | | **Bicalutamide** | | | **GnRH** | | **None** | | | |  |
|  | |  | Added treatment | |  | Added treatment |  | Added treatment | | | |
| Time to relapse | Gleason | N men | GnRH % | ARPI % | N men | ARPI  % | N men | Bicalutamide % | GnRH % | ARPI % | |
| Below 18 months | 6 | 5 | 40 | 0 | 5 | 0 | 67 | 16 | 15 | 0 | |
|  | 7 (3+4) | 7 | 43 | 0 | 12 | 0 | 60 | 25 | 12 | 0 | |
|  | 7 (4+3) | 10 | 40 | 0 | 13 | 0 | 72 | 35 | 24 | 0 | |
|  | 8 | 14 | 50 | 0 | 20 | 0 | 41 | 37 | 32 | 0 | |
|  | 9-10 | 18 | 72 | 0 | 31 | 3 | 24 | 46 | 42 | 0 | |
| 18-36 months | 6 | ≤5 | 0 | 0 | 0 | 0 | 60 | 15 | 8 | 0 | |
|  | 7 (3+4) | ≤5 | 50 | 0 | 0 | 0 | 85 | 29 | 9 | 0 | |
|  | 7 (4+3) | 7 | 14 | 0 | ≤5 | 0 | 86 | 34 | 14 | 0 | |
|  | 8 | 10 | 40 | 0 | 6 | 0 | 67 | 24 | 21 | 0 | |
|  | 9-10 | 15 | 53 | 0 | ≤5 | 0 | 47 | 28 | 32 | 0 | |
| Above 36 months | 6 | ≤5 | 0 | 0 | 0 | 0 | 73 | 19 | 7 | 0 | |
|  | 7 (3+4) | ≤5 | 0 | 0 | ≤5 | 0 | 107 | 24 | 1 | 0 | |
|  | 7 (4+3) | ≤5 | 0 | 50 | 0 | 0 | 102 | 30 | 3 | 0 | |
|  | 8 | ≤5 | 50 | 0 | 0 | 0 | 60 | 35 | 12 | 0 | |
|  | 9-10 | ≤5 | 100 | 0 | 0 | 0 | 55 | 35 | 18 | 0 | |

Supplementary Table 2. Proportion of treatment within 180 days after relapse in men who experienced a relapse after primary radiotherapy according to time to relapse, Gleason at biopsy, type of adjuvant ADT and calendar period of relapse.

| **Relapse in 2016-2024** | | | | | | | | | | | |
| --- | --- | --- | --- | --- | --- | --- | --- | --- | --- | --- | --- |
| **Type of adjuvant ADT** | | **Bicalutamide** | | | **GnRH** | | **None** | | | |  |
|  | |  | Added treatment | |  | Added treatment |  | Added treatment | | | |
| Time to relapse | Gleason | N men | GnRH % | ARPI % | N men | ARPI  % | N men | Bicalutamide % | GnRH % | ARPI % | |
| Below 18 months | 6 | 2 | 50 | 0 | ≤5 | 50 | 38 | 8 | 3 | 3 | |
|  | 7 (3+4) | 7 | 43 | 0 | 6 | 50 | 103 | 17 | 7 | 4 | |
|  | 7 (4+3) | 20 | 65 | 10 | 13 | 46 | 43 | 12 | 16 | 12 | |
|  | 8 | 27 | 44 | 11 | 12 | 75 | 14 | 36 | 7 | 7 | |
|  | 9-10 | 67 | 57 | 22 | 55 | 60 | 9 | 22 | 22 | 33 | |
| 18-36 months | 6 | 9 | 22 | 0 | 0 | - | 38 | 26 | 5 | 5 | |
|  | 7 (3+4) | 15 | 20 | 0 | ≤5 | 50 | 128 | 29 | 14 | 4 | |
|  | 7 (4+3) | 33 | 30 | 6 | ≤5 | 33 | 99 | 33 | 13 | 5 | |
|  | 8 | 43 | 33 | 7 | 8 | 25 | 33 | 42 | 27 | 0 | |
|  | 9-10 | 125 | 42 | 10 | 23 | 30 | 67 | 40 | 21 | 12 | |
| Above 36 months | 6 | ≤5 | 0 | 0 | ≤5 | 100 | 274 | 18 | 5 | 1 | |
|  | 7 (3+4) | 6 | 33 | 0 | 0 | - | 470 | 26 | 4 | 3 | |
|  | 7 (4+3) | 11 | 55 | 9 | ≤5 | 0 | 419 | 27 | 10 | 2 | |
|  | 8 | 15 | 27 | 7 | ≤5 | 67 | 229 | 35 | 10 | 4 | |
|  | 9-10 | 31 | 26 | 6 | 15 | 27 | 302 | 46 | 14 | 7 | |

GnRH=Gonadotropin-Releasing Hormone (or orchiectomy) +/- bicalutamide

ARPI=Androgen Receptor Pathway Inhibitor +/- GnRH +/- bicalutamide

Supplementary Table 3. Sensitivity analyses.

|  | **Sensitivity analysis** | | | | **Main analysis** |
| --- | --- | --- | --- | --- | --- |
|  | **1** | **2** | **3** | **4** |  |
| Description | Nadir cut-off 1 ng/ml if on ADT (else cut-off 2 ng/ml), prostatitis accounted for | ADT window of 365 days, prostatitis accounted for | ADT window 90 days, prostatitis accounted for | Ignore prostatitis | Nadir cut-off 2 ng/ml, ADT window of 189 days, prostatitis accounted for |
| **Outcomes in analysis of incidence of relapse** | **10-year risk  (%, 95% CI)** | **10-year risk  (%, 95% CI)** | **10-year risk  (%, 95% CI)** | **10-year risk  (%, 95% CI)** | **10-year risk  (%, 95% CI)** |
| Low-risk relapse | 14 (14-14) | 14 (14-14) | 14 (13-14) | 14 (14-14) | 14 (13-14) |
| High-risk relapse | 11 (11-11) | 11 (11-11) | 10 (10-11) | 11 (11-11) | 11 (11-11) |
| Salvage treatment | 3 (3-3) | 2 (2-2) | 4 (4-4) | 3 (3-3) | 3 (3-3) |
| Death from any cause | 2 (2-2) | 2 (2-2) | 2 (2-2) | 2 (2-2) | 2 (2-2) |
|  | **N (%)** | **N (%)** | **N (%)** | **N (%)** | **N (%)** |
| **Number of men with relapse** | 4132 (16) | 4056 (15) | 3969 (15) | 4053 (15) | 4024 (15) |
| **Outcomes in analysis of risk of death after relapse** | **10-year risk  (%, 95% CI)** | **10-year risk  (%, 95% CI)** | **10-year risk  (%, 95% CI)** | **10-year risk  (%, 95% CI)** | **10-year risk  (%, 95% CI)** |
| Risk of death from prostate cancer after relapse | 34 (32-36) | 35 (33-37) | 35 (33-36) | 35 (33-37) | 35 (33-37) |
| Risk of death from other causes after relapse | 30 (28-31) | 30 (28-31) | 30 (28-31) | 30 (28-31) | 30 (28-31) |

**Abbreviations:** CI=Confidence interval. ADT= Androgen deprivation therapy.

### References

1. ” Basic Clinical Radiobiology" (5th edition)”, Joiner, M. & van der Kogel, A. (2018).

2. Bentzen SM, Dörr W, Gahbauer R, Howell RW, Joiner MC, Jones B, et al. Bioeffect modeling and equieffective dose concepts in radiation oncology--terminology, quantities and units. Radiother Oncol. 2012 Nov;105(2):266–8.

3. Vogelius IR, Bentzen SM. Dose Response and Fractionation Sensitivity of Prostate Cancer After External Beam Radiation Therapy: A Meta-analysis of Randomized Trials. Int J Radiat Oncol Biol Phys. 2018 Mar 15;100(4):858–65.
